# Extending the Concept of Vaccinology to the Control of Multidrug-resistant Sepsis in Neonates

**DOI:** 10.7759/cureus.3077

**Published:** 2018-07-31

**Authors:** Karthikeyan Gengaimuthu

**Affiliations:** 1 Neonatology and Pediatrics, International Modern Hospital, Dubai, ARE

**Keywords:** probiotic, neonate, cross colonization, sepsis

## Abstract

Standard infection control bundles have not been consistently effective in combating sepsis due to multidrug-resistant organisms (MDROs). Recent trials showing the beneficial effects of probiotics in controlling late-onset sepsis, the so-called “cross-contamination” or “cross-colonization” phenomenon that draws a parallel with the herd immunity concept in vaccinology. This editorial highlights the putative benefits of adapting the vaccinology-based concept using probiotic bacteria in our combat against MDROs.

## Editorial

Neonatal host defenses against pathogens are deficient in both the general innate and the specialized adaptive immune responses, (e.g., complement and macrophage systems, and T-cells and B-cells, respectively) [1]. Therefore, sepsis readily overwhelms these vulnerable young infants, including the early onset of systemic symptoms that are often non-specific and is an important determinant of mortality [1-2]. Infections with multidrug-resistant organisms (MDROs) in neonatal units are becoming increasingly common and available therapeutic options are limited, especially in resource-poor settings.

Combating neonatal sepsis: a vaccinology-based approach

Passive strategies, like the administration of intravenous (IV) immunoglobulin, have a strong theoretical basis yet consistently fail when tested in vivo (International Immunoglobulin Study (INIS), 2014) [1]. The PROphylactic GRAnulocyte Macrophage colony-stimulating factor to reduce sepsis in preterm neonates (PROGRAMs) trial with granulocyte-macrophage–colony stimulating factor (GM-CSF) also failed to reduce either the incidence or the mortality due to sepsis despite a documented booster effect in neutrophil defenses [2]. An active strategy has yet to be actively pursued and realized, and this is most likely due to concerns regarding the immune incompetency of the neonates and the time frame required to achieve the protective levels of specific immune effectors.

The cross-contamination phenomenon: serendipitous observations from probiotic trials

Two, recent, well-designed, randomized, placebo-controlled trials (Probiotics in Preterm Infants Study (PiPS) in the UK and the ProPrems study in Australia] on the use of probiotic in neonates documented evidence of the cross-contamination phenomenon (colonization of the gut with probiotics in the control group neonates) [3]. This phenomenon occurred in 49% of the control group in the PiPS study while the Australian study referred to its likely contribution to their observed outcome in the discussion section of their published article. We have since published a hypothetical reanalysis of the PiPS trial that revealed statistically significant benefits conferred by the probiotic Bifidobacterium breve in terms of the reduced occurrence of necrotizing enterocolitis, late-onset sepsis, and the all-cause mortality when this significant confounding factor of cross-contamination was eliminated. We have also analyzed hypothetically the putative mechanisms underlying this cross-contamination phenomenon as well as its potential applications in our recently published work in this journal [3].

Cross-thoughts on the cross-colonization phenomenon

The colonization of the gut with gram-negative enteric bacteria and the consequent translocation is the most common culprit in the causation of bloodstream infections in neonates. In an Italian study, biweekly rectal swabs were collected to determine the levels of multidrug-resistant gram-negative bacilli. They found that 55% of the neonates were colonized and 72% of these were due to cross-colonization, as proven by molecular analysis [3]. The putative benefit that the probiotics offer in reducing the late-onset sepsis in neonates probably accrue from their proven ability to achieve gut colonization and cross-colonization.

Preventing MDRO sepsis in neonates: a vaccinology-based strategy

"A thorn must be removed by another thorn" so goes an Indian language proverb. This proverb aptly describes the cross-colonization phenomenon's ability to prevent the pathogenic colonization of bacteria. More importantly, this can be the basis of a vaccinology-based therapeutic strategy, which, in turn, can be effectively deployed to achieve our objective of preventing MDRO sepsis in neonates. The basic premise of vaccinology-based therapeutics is that gut colonization with gram-negative bacteria and surface colonization with beneficial probiotic bacteria confers protection against bloodstream infections with MDROs. The key is to achieve rapid colonization and the quicker this is achieved, the higher the protective efficacy of this strategy will be. Traditional probiotics need three to five days to achieve gut colonization and, hence, may not prevent the rapid dissemination of MDRO sepsis observed in neonatal units during outbreaks. The next key concept is achieving long-term colonization with single or limited doses rather than the continuous daily administration that is currently required with the available probiotics. Recombinant vaccine development technology using virulent gene arrays and the proteomics of MDROs may help us further in the development of candidate probiotic bacteria to achieve this objective.

 A study published in the journal Nature Reports that Lactobacillus plantarum American type culture collection (ATCC) strain 202195 in combination with fructooligosaccharide achieved colonization for up to four months, significantly reduced infections with gram-positive and gram-negative bacteria, and decreased respiratory infections in rural India [4]. A novel gram-negative probiotic bacterium E. coli Nissle 1917 that has a persistent gut colonization effect can be an effective single-dose weapon in our fight against MDR gram-negative infections [5].

It may be concluded that probiotic bacteria can effectively compete with and eliminate colonization with virulent pathogens, including MDROs, and further research is needed to identify the unique probiotic strains that could achieve this.
